# Computational Analysis in Laminar Flow of Several Nanocolloids with PEG 200 and MgO/MWCNTs Nanoparticles

**DOI:** 10.3390/ma19081617

**Published:** 2026-04-17

**Authors:** Alina Adriana Minea, Catalin Andrei Tugui, George Catalin Tofan, Elena Ionela Chereches

**Affiliations:** Faculty of Materials Science and Engineering, Technical University Gheorghe Asachi Iasi, Bd. D. Mangeron 63, 700050 Iasi, Romania; george-catalin.tofan@student.tuiasi.ro (G.C.T.); elena-ionela.chereches@academic.tuiasi.ro (E.I.C.)

**Keywords:** nanocolloids, PEG 400, heat transfer coefficient, laminar flow, PEG 200

## Abstract

**Highlights:**

- A comparative numerical analysis of different PEG nanocolloids was performed.
- Chemicals thermophysical properties were experimentally assessed.
- The heat transfer coefficient was evaluated and comparatively discussed.
- A comparison of modeling approaches was discussed.

**Abstract:**

This study presents a numerical investigation of the laminar forced convection of polyethylene glycol-based nanocolloids within a horizontal pipe. To bridge the gap between theoretical predictions and practical performance, simulations were conducted over a Reynolds number range of 500 to 2000, utilizing a model validated against laboratory-scale experimental data and well-defined boundary conditions. Our analysis focuses on the thermal behavior of polyethylene glycol 200 enriched with metal oxide nanoparticles and multi-walled carbon nanotubes, which were selected for their capacity to enhance thermal conductivity while maintaining manageable viscosity. The results demonstrate that PEG 200-based nanocolloids significantly improve heat transfer performance in the laminar regime. This enhancement is attributed to the superior intrinsic thermal properties of the nanoparticles and the complex synergistic interactions—such as Brownian motion and thermophoresis—between the particles and the PEG base fluid. A critical evaluation of the standard approach of incorporating thermophysical properties into the numerical approach led to significant discrepancies in flow predictions. Additionally, our study establishes that assuming constant thermophysical properties during the heating process introduces simulation errors exceeding 10%. These findings underscore the necessity of incorporating temperature-dependent, experimentally validated data into numerical models to ensure predictive accuracy. Ultimately, this work advocates for a nuanced approach to nanocolloid design that prioritizes the specific chemical and rheological compatibility between nanoparticle types and the base fluid.

## 1. Introduction

Nanocolloids have been extensively investigated as an advanced solution for enhancing heat transfer in energy and industrial applications because it is possible to modify their base fluid’s thermophysical properties through the addition of nanoparticles. In this context, numerical CFD (computational fluid dynamics) simulations play a crucial role in understanding flow and heat transfer mechanisms, enabling a detailed analysis of the influence of nanoparticle type on the overall behavior of the nanocolloid. Although the literature reports numerous numerical studies on water- or ethylene glycol-based nanocolloids, the use of polyethylene glycol (PEG) as a base fluid remains limited, particularly in the context of numerical modeling, despite its favorable rheological and thermal properties.

PEG 200 (i.e., a liquid polyethylene glycol with molecular mass of 200 g/mol) is characterized by its high chemical stability, low vapor pressure, compatibility with a wide range of materials, and advantageous rheological behavior at moderate temperatures, which makes it suitable for applications where conventional base fluids may exhibit limitations. Nevertheless, the complexity of its thermophysical properties and the lack of generally accepted correlations for PEG-based nanocolloids have led to its limited representation in CFD numerical studies compared to classical base fluids.

Moreover, MgO and multi-walled carbon nanotube (MWCNT) nanoparticles exhibit distinct heat transfer enhancement mechanisms—ranging from high thermal conductivity and chemical stability to percolation effects and anisotropy—yet their comparative impact within a PEG 200-based nanocolloid has not been systematically analyzed using CFD methods, which motivates and defines the scope of the present numerical study [1].

In the following paragraphs, the main types of nanocolloids investigated in the literature through CFD-based numerical simulations are presented and critically reviewed, with particular emphasis on studies employing MgO and MWCNT nanoparticles, as well as other relevant nanocolloid systems, in order to highlight current trends, modeling approaches, and existing gaps in numerical research.

Ding et al. [2] demonstrated that multi-walled carbon nanotube-based nanocolloids can enhance convective heat transfer by over 350% at Re = 800 and concentrations of 0.5 %wt., an effect attributed to shear-thinning behavior and enhanced thermal conduction mechanisms. Davarnejad and Jamshidzadeh [3] investigated the turbulent heat transfer behavior of magnesium oxide-water nanocolloids in circular tubes using CFD simulations (Re = 3000–19,000, volume fractions 0.0625–1%), demonstrating that two-phase models (VOF and mixture) exhibit superior accuracy compared to the single-phase model, with the Nusselt number increasing with nanoparticle concentration, while the friction factor increase was negligible. Ko et al. [4] experimentally investigated the flow characteristics of aqueous carbon nanotube suspensions. Comparing two stabilization methods (surfactant and acid treatment), they demonstrated shear-thinning behavior for both nanocolloid types, with friction factors higher than distilled water under laminar flow but similar to the base fluid under turbulent flow, and showed that increased CNT loading extends the laminar regime to higher flow rates, enabling lower friction factors than pure water at certain flow rate ranges. Meyer et al. [5] experimentally investigated the convective heat transfer of aqueous multi-walled carbon nanotube suspensions in late laminar, transitional, and early turbulent flow regimes (Re = 1000–8000, volume concentrations 0.33–1.0%), demonstrating that although nanocolloids show apparent enhancement on Nu–Re plots, at the same fluid velocity, the heat transfer coefficient decreases compared to water due to increased viscosity. They concluded that the viscosity increase exceeds the thermal conductivity enhancement by a multiple of four, resulting in an inefficient nanocolloid for heat transfer applications. Gupta et al. [6] experimentally investigated the convective heat transfer of MWCNT/water nanocolloids in laminar flow through uniformly heated copper tubes (concentrations 0.05–0.5 %wt., velocities 0.166–0.232 m/s), employing constant velocity criteria for accurate comparison, and achieved a maximum heat transfer coefficient enhancement of 77.60% at 0.5 %wt. concentration and 0.232 m/s velocity compared to distilled water.

Recent studies, such as those by Demirpolat et al. [7], have demonstrated that MgO-based nanocolloids significantly enhance convective heat transfer coefficients in laminar in-pipe flows, highlighting their strong potential for improving thermal performance in energy systems.

Zhang et al. [8] demonstrated that increasing the volume concentration of water-based MgO nanocolloids in a shell-and-tube heat exchanger enhances heat transfer performance, with peak improvements of 14.52% in the overall heat transfer coefficient and 13.02% in the Nusselt number at a 0.35% concentration, while numerical simulations validated by experiments confirmed reliable predictions of thermal and hydraulic parameters. Kumar et al. [9] established that combining a Diamond Fin Vortex Generator with a hybrid MWCNT-MgO nanocolloid significantly enhances natural convective heat transfer in horizontal elliptical annuli. Achieving an up to 28% higher convective heat transfer coefficient, a 15% increase in the Nusselt number, and reduced surface temperatures, they highlighted the potential of hybrid nanocolloids and flow disruption techniques to improve thermal management in industrial cooling systems. Cardenas Contreras and Bandarra Filho [10] investigated the heat transfer performance of MWCNT nanocolloids in a 50:50 water–ethylene glycol mixture within an automotive radiator, showing that nanoparticle addition can improve the heat transfer rate and overall heat transfer coefficient by up to 4.6% and 4.4%, respectively, while highlighting the challenges of nanoparticle stability and performance reduction at high inlet temperatures. Highlighting the potential of MWCNTs for solar thermal applications, Elshazly et al. [11] demonstrated that a 0.5% MWCNT/water nanocolloid can enhance the energy and exergy efficiency of evacuated tube solar collectors by up to 73.5% and 51%, respectively, outperforming conventional Al_2_O_3_ nanocolloids. Said et al. [12] showed that MWCNT/water nanocolloids significantly enhance shell-and-tube heat exchanger performance. These nanocolloids achieved up to a 31.08% increase in the heat transfer coefficient and a 15.4% improvement in overall effectiveness when combined with semicircular baffles, and validated numerical and AI-based models confirmed their techno-economic and environmental viability.

In the framework of PEG heat transfer fluids, Cojocariu et al. [13] demonstrated that polyethylene glycol-based fluids (PEG 200 and PEG 400) and their mixtures with water exhibit thermal effusivity strongly dependent on molar mass and composition, with water-rich mixtures enhancing heat transfer capacity. At the same time, they highlighted nanoparticle addition to PEG-based fluids as a promising pathway for further thermal performance improvement. In addition, Minea et al. [14] numerically investigated the hydrothermal performance of a newly developed polyethylene glycol (PEG 400)-based nanocolloid with ZnO nanoparticles for HVAC applications. Using experimentally determined thermophysical properties at various concentrations (0.5–5%) and temperatures for Re = 200–2000, they demonstrated heat transfer enhancements up to 16% with a 13% pressure drop penalty and improved performance evaluation criteria, at the same time developing correlations for the Nusselt number and friction factor as functions of operating conditions. Veeram et al. [15] numerically investigated (bvp4c MATLAB solver) the radiative flow of PEG- and water-based hybrid nanocolloid with ZrO_2_ and MgO nanoparticles over a curved shrinking sheet with viscous dissipation and a higher-order chemical reaction, comparing performance with mono-nanocolloids (PEG–water + ZrO_2_). They demonstrated that hybrid nanocolloids offer superior heat transmission capabilities, with skin friction coefficient increases of 348.1% (hybrid) versus 274.6% (mono) at volume fractions 0–0.2%, confirming hybrid nanocolloids’ superiority for applications in heat exchangers, solar collectors, and electronic cooling. Cherecheș et al. [16] performed a numerical investigation of the thermal transfer performance of PEG 400-based nanocolloids containing Al_2_O_3_ and ZnO nanoparticles, showing that ZnO-based nanocolloids exhibit superior heat transfer enhancement due to their higher thermal conductivity, and that the heat transfer coefficient increases with both nanoparticle concentration and the Reynolds number.

Recently, PEG 400 and PEG 200 have been discussed in terms of their behavior in thermal applications by Tofan et al. [17] and Minea et al. [18]. The analyses included several PEC (performance evaluation criteria) that revealed that the addition of MWCNTs to PEG decreases the thermal transport. Plus, MWCNT nanocolloids indicate a pumping power increase of up to 29.7%, depending on the nanoparticles loading.

In reviewing the current state of research and the studies reported by the aforementioned authors, we identified no dedicated numerical investigations of PEG 200-based nanocolloids, although there are a few studies on PEG 400-based nanocolloids, a polyethylene glycol of the same category. This constitutes a gap in the literature. To bridge the gap in nanocolloid research, this paper presents a numerical investigation of the convective heat transfer of MgO and MWCNT nanoparticles suspended in PEG 200. Unlike generalized models, this study utilizes boundary conditions and thermophysical data derived directly from experimental measurements. The primary contribution is twofold: the rigorous validation of the numerical framework and the focus on the distinct thermal behavior of PEG 200 nanocolloids. By analyzing the flow across 500 < Re < 2000 and temperature-dependent properties, this work provides essential benchmarks for realistic heat transfer modeling.

## 2. Methodology

### 2.1. Chemicals and Thermophysical Properties: Experimental Approach

All the chemicals were acquired from Sigma-Aldrich (St. Louis, MO, USA), and their intrinsic properties, as per manufacturer, are described in Table 1. The nanocolloids were manufactured by the two-step method, in concentrations up to 2.5 %wt. MgO and 0.3 %wt. MWCNT. The procedure followed a strict protocol, described in detail by Cojocariu et al. [19], and the samples’ stability was checked through several methods—visualization, PH and PDI (polydispersity index)—as was described in this group’s previous experimental data analysis (see [19,20]).

The thermophysical properties were determined employing several pieces of equipment that are available in the lab, including the IKA viscosimeter (IKA-Werke GmbH & Co. KG, Staufen, Germany); the C-Therm (C-Therm Technologies Ltd., Fredericton, NB, Canada), for thermal conductivity and effusivity; as well as the Digital Densimeter DS7800 (A.KRÜSS Optronic GmbH, Hamburg, Germany). The entire procedure and a discussion of the results have been published in earlier reports (please see the details in Cojocariu et al. [19,20]). Regarding the viscosity tests, the data were collected at a shear rate of 10.56 1/s (i.e., corresponding to 8 RPM) to guarantee a torque value between 10 and 90% regardless of the increasing temperature (see more details about the experimental procedure in [19,20]).

**Figure 1:**
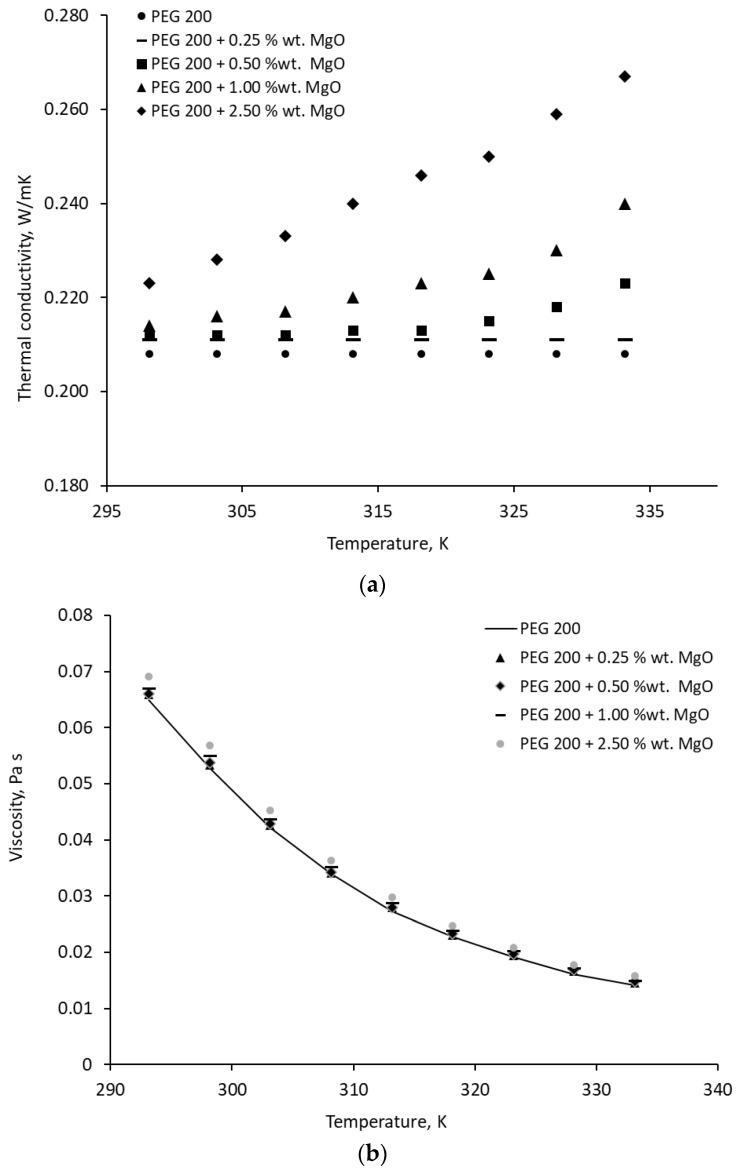
Viscosity and thermal conductivity variation with temperature for MgO − PEG 200 nanocolloids: (**a**) thermal conductivity variation with temperature; (**b**) viscosity variation with temperature.

**Figure 2:**
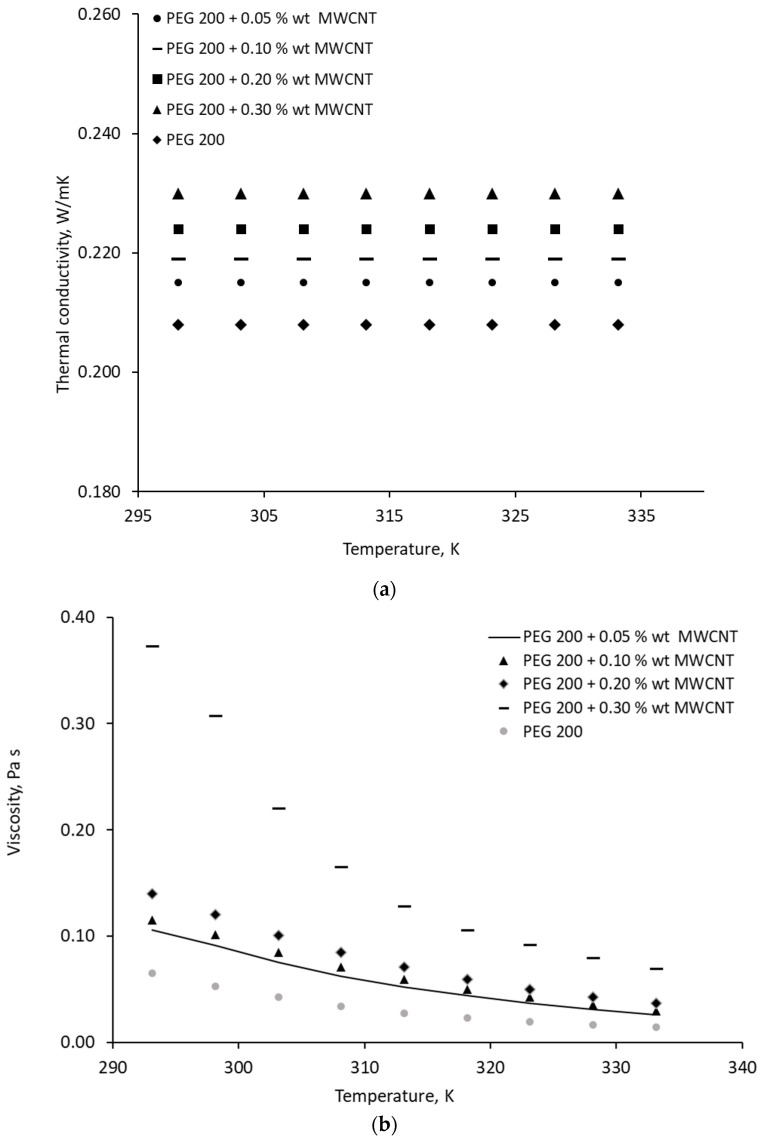
Thermal conductivity (**a**) and viscosity (**b**) variation with temperature for MWCNT − PEG 200 nanocolloids.

Some of the experimental results for MgO nanocolloids and MWCNTs are presented in Figure 1 and Figure 2, respectively.

From Figure 2a, one can see that the addition of low percentages of MWCNTs does not influence the thermal conductivity’s variation with temperature, but that the thermal conductivity does increases with MWCNT percentage. Viscosity, on the other hand, increases up to 0.38 Pa s at ambient temperature, which greatly impacts the heat transfer, especially at MWCNT concentration exceeding 0.2 %wt.

All the properties were implemented in the Ansys 2025 R1 CFD code [21].

### 2.2. Numerical Approach

The numerical analysis examines steady, laminar forced convection of nanocolloids inside a horizontal tube with a diameter of 0.12 m and an overall length of 8.64 m. The tube was divided into two sections: an initial isothermal region measuring 5.76 m, followed by a uniformly heated segment of 2.88 m. The inlet temperature of the fluid was maintained at 300 K, and the simulations were performed for Reynolds numbers between 500 and 2000. A constant heat flux of 8000 W/m^2^ was imposed on the tube wall in the heated (downstream) section (see [1,17] for details on the geometry and boundary conditions).

The heat transfer characteristics of the nanocolloids were modeled using the governing continuity, momentum, and energy equations, which are presented in Equations (1)–(4) and detailed in Minea [1]. The numerical simulations were conducted with the ANSYS Fluent Workbench 2025 v1 [21]. Additional details regarding the laminar flow model and the numerical methodology are available in [1,22].

Continuity equation:

(1) $$\frac{1}{R}\frac{\partial }{\partial \theta }(\rho_{nf}U)+\frac{1}{R}\frac{\partial }{\partial R}(\rho_{nf}RV)+\frac{\partial }{\partial z}(\rho_{nf}W)=0.$$

Momentum equation on R direction:

(2) $$\begin{array}{l} \frac{1}{R}\frac{\partial }{\partial \theta }(\rho_{nf}UV)+\frac{1}{R}\frac{\partial }{\partial R}(\rho_{nf}RVV)+\frac{\partial }{\partial z}(\rho_{nf}WV)-\frac{1}{R}(\rho_{nf}U^{2})=\\ =-\frac{1}{R}\frac{\partial P}{\partial \theta }+\frac{1}{R^{2}}\frac{\partial }{\partial \theta }(\mu_{nf}\frac{\partial V}{\partial \theta })+\frac{\partial }{\partial R}(\frac{\mu_{nf}}{R}\frac{\partial (RV)}{\partial R})-\frac{2\mu_{nf}}{R^{2}}\frac{\partial U}{\partial \theta } \end{array}.$$

Energy equation:

(3) $$\begin{array}{l} \frac{1}{R}\frac{\partial }{\partial \theta }(\rho_{nf}U\Theta )+\frac{1}{R}\frac{\partial }{\partial R}(\rho_{nf}RV\Theta )+\frac{\partial }{\partial z}(\rho_{nf}W\Theta )=\\ =\frac{1}{R^{2}}\frac{\partial }{\partial \theta }\left(\frac{k_{nf}}{c_{pnf}}\frac{\partial \Theta }{\partial \theta }\right)+\frac{\partial }{R\partial R}\left(R\frac{k_{nf}}{c_{pnf}}\frac{\partial \Theta }{\partial R}\right) \end{array}.$$

where the nondimensional variables are defined as:

(4) $$R=\frac{r}{D},Z=\frac{z}{D},U=\frac{u}{u_{\infty }},V=\frac{v}{u_{\infty }},W=\frac{w}{u_{\infty }},\Theta =\frac{T-T_{\infty }}{T_{W}-T_{\infty }},P=\frac{p}{\rho u_{\infty }^{2}}.$$

In these equations, ρ_nf_, μ_nf_, C_nf_, and k_nf_ denote the density, dynamic viscosity, specific heat capacity, and thermal conductivity, respectively, of the nanocolloids. For comparison purposes, additional simulations were conducted in which the thermophysical properties of the nanocolloids were replaced by those of the base fluid, allowing the reference flow behavior to be evaluated under identical operating conditions.

The governing equations were discretized using the finite volume approach, which entailed converting the partial differential equations into a system of algebraic equations solvable through numerical methods. A second-order upwind discretization scheme was applied to both convective and diffusive terms to improve solution accuracy. Pressure–velocity coupling was handled using the SIMPLE (Semi-Implicit Method for Pressure-Linked Equations) algorithm, which is appropriate for incompressible laminar flow regimes [23,24].

At the inlet of the tube, a fully developed velocity profile was specified for all working fluids to ensure uniform hydrodynamic boundary conditions across the simulations.

#### Boundary Conditions and Validation

The flow entering the test section was assumed to be hydrodynamically developed with a uniform inlet temperature, and a constant wall heat flux (i.e., of 8000 W/m^2^) was applied along the heated section of the pipe.

Momentum conservation conditions included a fully developed velocity profile at the inlet, a no-slip condition at the tube wall, and a constant static pressure at the outlet, where the reference (relative) pressure was set to zero.

Energy conservation conditions consisted of a prescribed constant temperature at the inlet and a specified heat flux at the wall. No thermal boundary condition was imposed at the outlet, where a convective outflow condition was adopted.

To assess grid independence, multiple mesh arrangements were examined. The final computational grid comprised 100 × 180 nodes, with 100 nodes in the radial direction and 180 nodes in the axial direction. This mesh was selected after observing that further refinement resulted in variations of less than 5% in predicted outlet velocity and temperature while substantially increasing computational cost. Additional mesh refinement was applied near the inlet and the tube wall to better capture steep temperature gradients. Differences in the predicted Nusselt number were found to be below 2%, supporting the use of the selected mesh for subsequent simulations.

**Figure 3:**
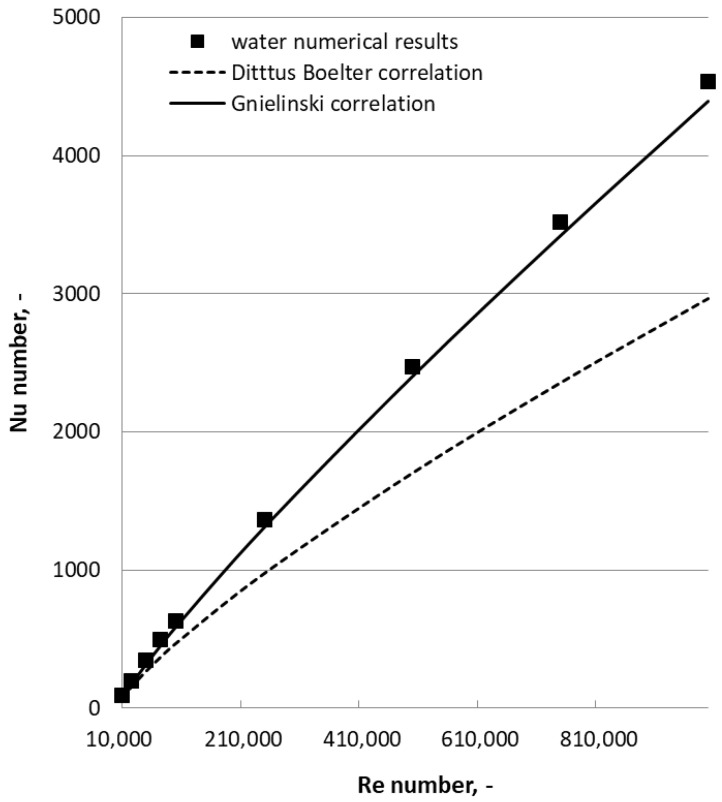
Model validation (see [17,23] for more insights).

Model validation was carried out by simulating pure water flow under the same conditions and comparing the results with reference data. As illustrated in Figure 3, the numerical results closely match theoretical values obtained from the Dittus–Boelter [25] and Gnielinski [26] correlations.

## 3. Results and Discussion

### 3.1. Preliminary Analysis

**Figure 4:**
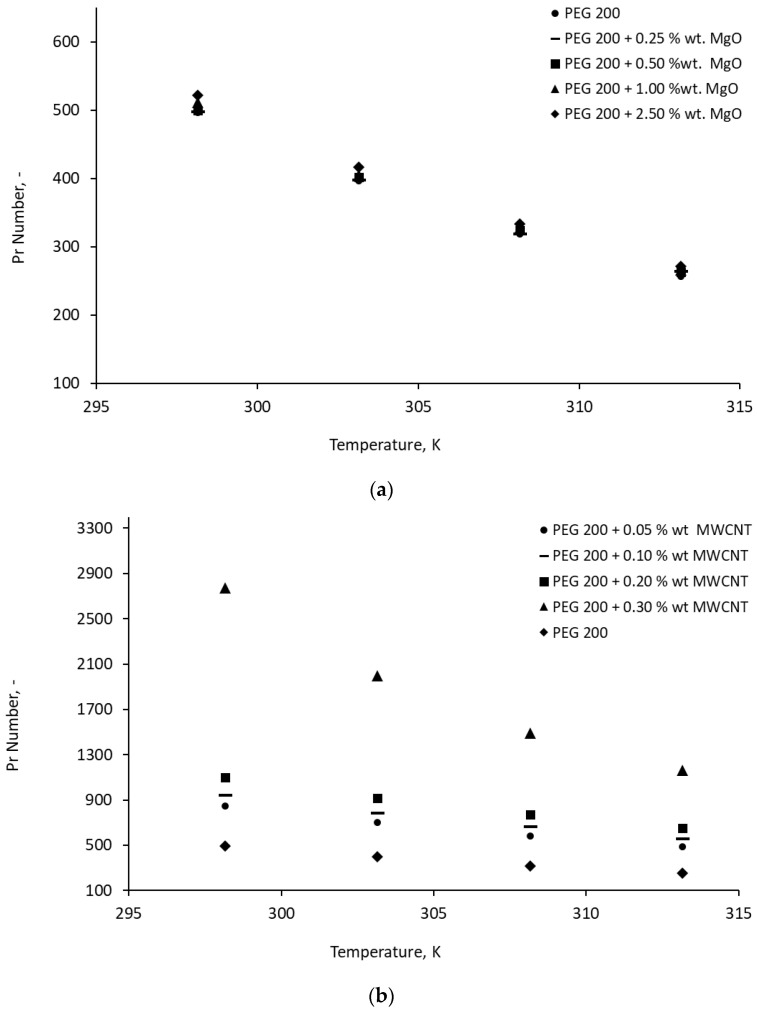
Pr number variation with temperature for the nanocolloids studied. (**a**) PEG 200 + MgO. (**b**) PEG 200 + MWCNT.

The CFD analysis was performed using the Prandtl number (as per Figure 4) and started with a comparison of the heat transfer coefficient for PEG 200 with constant properties and variable properties.

Figure 4 shows the Pr variation with temperature, nanoparticle loading, and nanocolloid type for all suspensions. Results indicate that Pr decreases with temperature and increases with NP addition. The largest increase is noticed for MWCNT nanocolloids due to the high viscosity of the suspensions (see Figure 2) combined with enhanced specific heat. An increased Prandtl number (Pr) indicates that kinematic viscosity dominates over thermal diffusivity, resulting in a thinner thermal boundary layer compared to the momentum boundary layer. This leads to reduced heat transfer rates, lower fluid temperatures away from the tube wall, and higher shear stresses at the wall.

**Figure 5:**
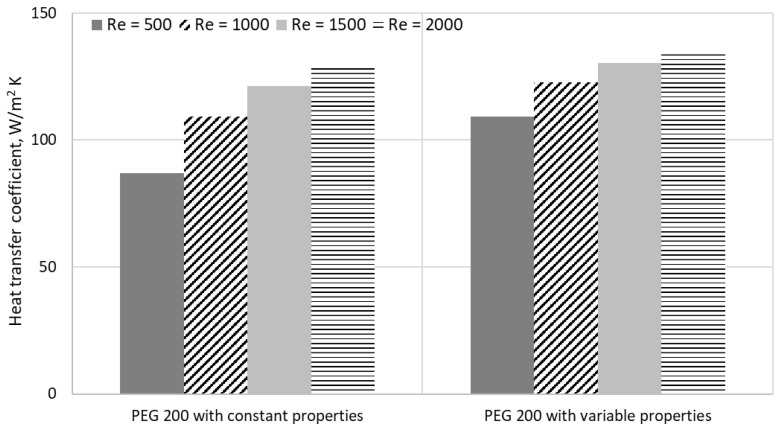
Comparison of heat transfer coefficient for properties implementation approach for PEG 200.

As was affirmed earlier, before incorporating all the thermophysical properties into the simulation, a comparative analysis in terms of approach was performed for PEG 200. More specifically, firstly it was assumed that all the properties remain constant for the studied temperature range (i.e., the assumption that is present in almost all published papers on numerical simulation for nanocolloids). Figure 5 outlines the comparison in terms of the heat transfer coefficient for the two cases considered and for different Re numbers.

As can be seen from the analysis, the differences lie between 5 and 26% and are higher for low Re numbers. This phenomenon outlines the relevance of considering the real variation of each thermophysical property. Consequently, we employed the experimental determined properties and their variation with temperature in the numerical analysis. This can clearly provide more accurate results, given that viscosity decreases with temperature, while specific heat and thermal conductivity are enhanced when temperature rises.

Thus, the analysis was performed considering the thermophysical properties variation with temperature for all suspensions.

### 3.2. Numerical Results Analysis

**Figure 6:**
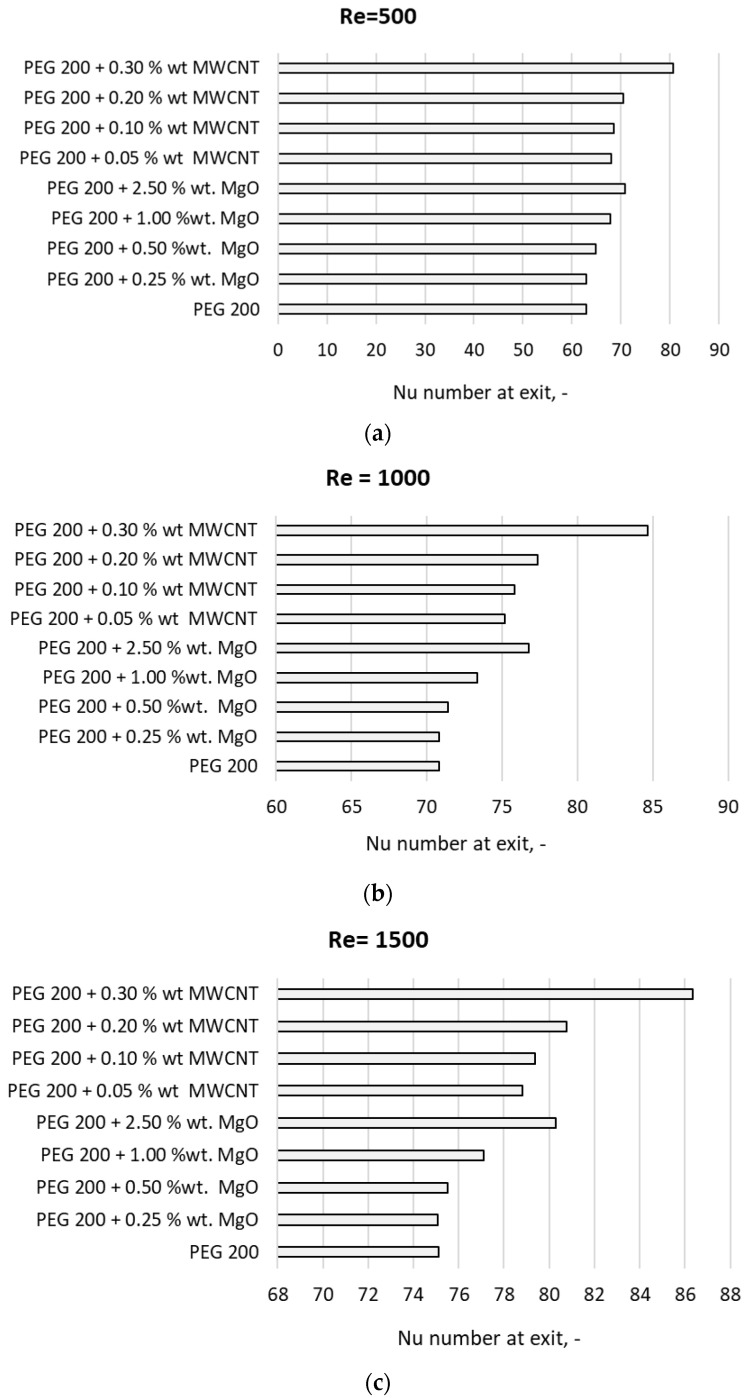

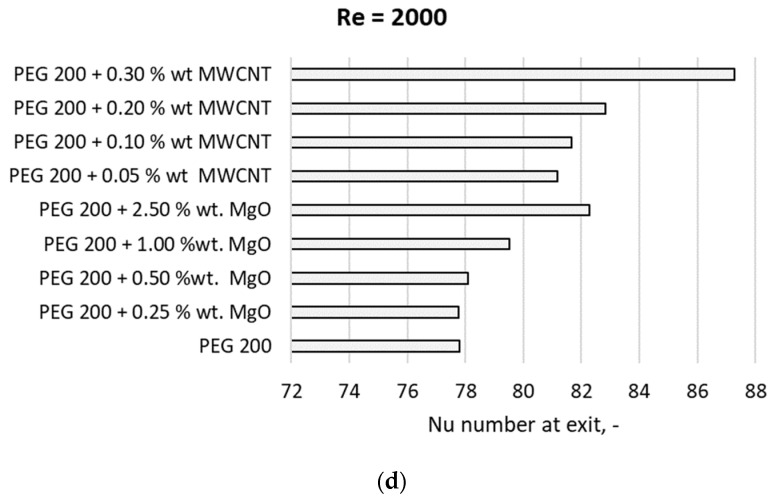
Numerical results for different Re numbers: (**a**) Re = 500; (**b**) Re = 1000; (**c**) Re = 1500; (**d**) Re = 2000.

The numerical analysis was performed for Re numbers ranging from 500 to 2000, and all the nanocolloids properties were implemented as experimentally determined. This technique assures the most reliable results, as was previously demonstrated in the large benchmark study conducted by Minea et al. [24]. Throughout the analysis, three parameters where monitored: the Nusselt number calculated from the Ansys code, medium temperature and wall temperature at exit. The results in terms of the Nusselt number at exit are provided in Figure 6, which shows the influence of each NP type and the NP concentration on the PEG 200 behavior in laminar flow. The increase in the Re number goes to an increase in Nu, and this increase is larger when the NP loading increases. This is a normal phenomenon observed for all nanocolloids’ flow in the laminar regime and occurs due to the increase in thermal conductivity, the main driving force in this situation.

**Figure 7:**
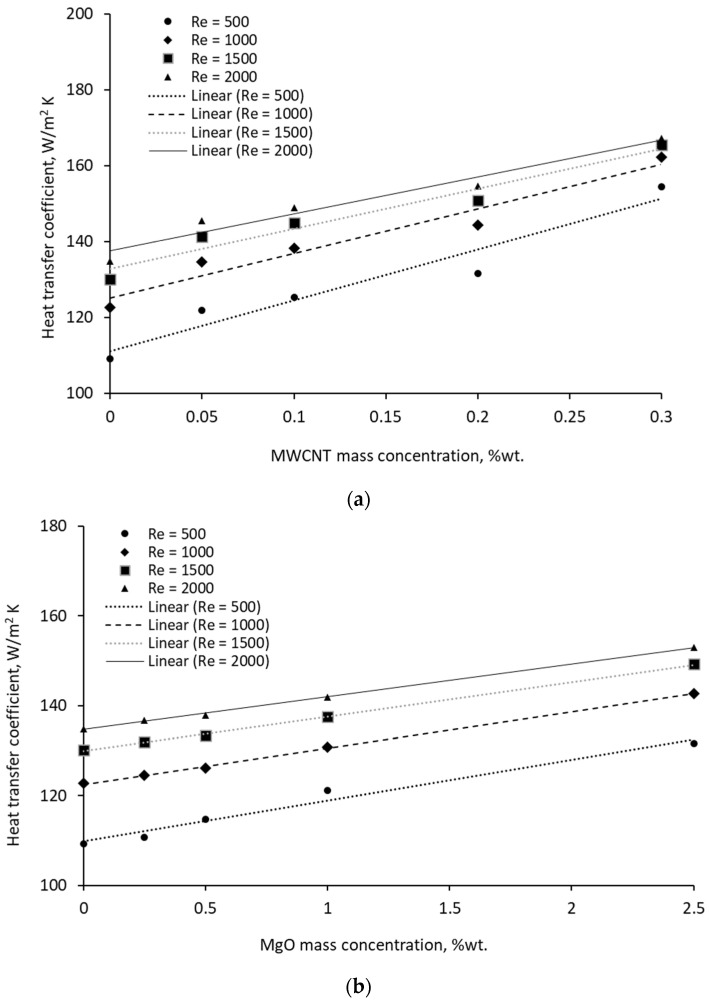
Variation of heat transfer coefficient with Re and NP mass concentration: (**a**) MWCNT suspensions. (**b**) MgO suspensions.

The heat transfer coefficient was calculated based on the monitored temperatures and tube geometry and was found to increase with temperature for each Re number, as is shown in Figure 7. The data analysis revealed that the heat transfer coefficient decreases with Re number, and this phenomenon occurs due to an increase in the viscosity of the nanocolloids, which results in low Re flows. Figure 7 shows a linear increase with nanoparticle concentration for each nanocolloid at a certain Re.

The variation can be estimated with a linear regression, which has a very good accuracy of over 95%, as is outlined in Table 2, where h is the heat transfer coefficient and x is the nanoparticle mass concentration. In addition, several statistical data were calculated based on the R-squared value, number of observations and predictors (i.e., number of independent variables). The small *p*-values, as estimated in Table 2, indicate that the regression is statistically significant.

**Figure 8:**
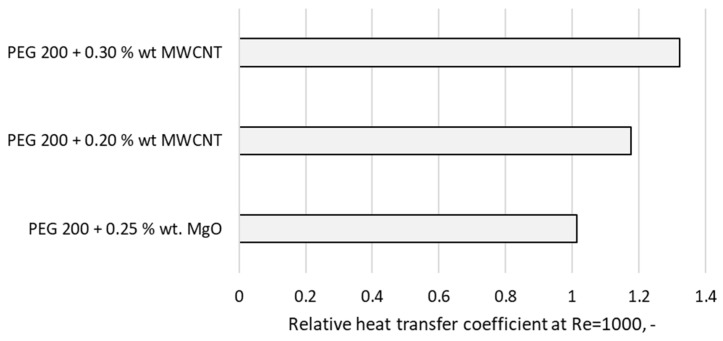
Comparison of heat transfer coefficient for MWCNT and MgO suspensions.

Additionally, a comparison of the relative heat transfer coefficient (defined as the ratio of the heat transfer coefficient of the nanofluid to that of the base fluid) was performed for similarly loaded suspensions, as shown in Figure 8. The numerical data at Re = 1000 indicate that the addition of MWCNTs is more effective in enhancing laminar heat transfer at low concentrations than MgO nanoparticles. This behavior is primarily attributed to the increased intrinsic thermal conductivity of the nanoparticles, which is the main driving force in laminar convection.

Numerical data for PEG 200-based nanocolloids show an increase in heat transfer when nanoparticles are added and Re increases, a phenomenon that is also noticed in the open literature [2,4,7]. The laminar flow is positively influenced by high conductive nanoparticles, and the increase depends on nanoparticle type and concentration. For PEG 200 + MgO nanocolloids and Re = 500, the increase is between 1.5 to 20.5%. A larger enhancement is noticed for 2.5 %wt. MgO. When Re increases, the heat transfer coefficient increase is lower (i.e., 13.4% for PEG 200 + 2.5 %wt. at Re = 2000). A similar phenomenon is noticed for suspensions with MWCNTs, where smaller concentrations result in a larger increase mainly due to the upsurge in thermal conductivity.

### 3.3. Comparison with the Literature

**Figure 9:**
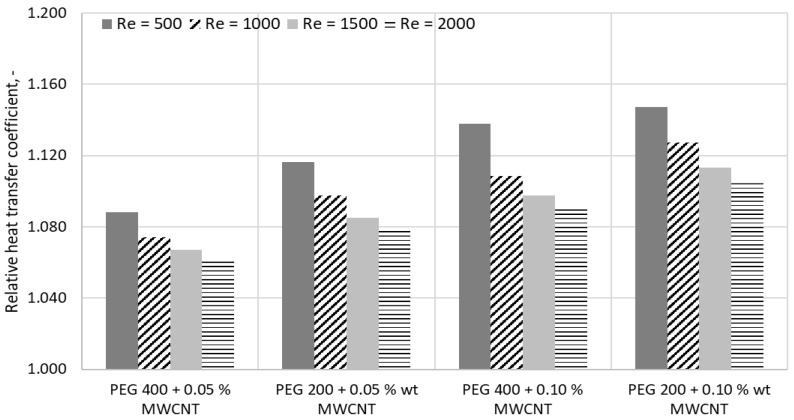
Relative heat transfer coefficient for different PEG + MWCNT nanocolloids: influence of the base fluid. Data is compared with previous results [17].

This section is dedicated to a comparison with our previous studies on PEG 400 with MWCNTs, as is illustrated in Figure 9. Our previous numerical approach involved a similar flow configuration and experimental thermophysical properties, as is discussed in Tofan et al. [17]. The data comparison revealed the importance of the base fluid due to its intrinsic properties (see Table 3 for the PEGs’ properties at ambient temperature).

Data from Figure 8 reveal a better behavior in laminar flow for PEG 200 compared with PEG 400, and this is due to the more favorable thermophysical properties of polyethylene glycol, which has a lower molecular mass. PEG 200 has an higher thermal conductivity (i.e., at around 10%) and a lower viscosity (i.e., about 52%) compared with PEG 400.

In conclusion, PEG 200, a low-molecular-weight liquid polymer, is non-toxic, biodegradable, and highly versatile as a base fluid. When converted into a nanocolloid (by dispersing nanoparticles like oxides or carbon nanotubes), its properties shift significantly, opening pathways for several technical applications in electronics cooling (dissipating heat from high-power microchips more efficiently than pure liquids), solar thermal collectors (adding carbon-based nanoparticles allows the fluid to absorb solar radiation directly, increasing the efficiency of solar water heaters), and thermal buffers (PEG 200 nanocolloids can be used to stabilize higher-molecular-weight PEGs).

## 4. Flow Analysis Based on Figures of Merit and Performance Evaluation Criteria

When discussing nanofluids (base fluids with suspended nanoparticles), figures of merit (FOMs) are used to evaluate whether the enhanced heat transfer justifies penalties like increased viscosity, pumping power, or cost. The key idea is always a trade-off between thermal enhancement and flow resistance. Adding nanoparticles typically increases thermal conductivity, improves convective heat transfer, increases viscosity (i.e., higher friction losses) and raises pumping power requirements. Thus, a nanofluid is only “better” if performance gains outweigh hydraulic and economic penalties.

Furthermore, a discussion of several factors that can better describe the flow performance is introduced in this section based on several well-known figures of merit.

Thus, a first figure of merit (FOM) that focuses on pressure loss (i.e., based on the $\frac{1}{\Delta P}$), FOM1, is defined as:

(5) $$FOM1=\;{\left(\Delta P\right)}_{bf}\;/\;{\left(\Delta P\right)}_{nf}\;\;$$

with ΔP described by the Darcy–Weisbach equation as:

(6) $$∆P=f\;\frac{L}{D}\;\frac{\rho v^{2}}{2}$$

where: f is the friction factor (depends on roughness & flow regime), L/D is the pipe length-to-diameter ratio and v is velocity.

Another well-known FOM is defined as the Mouromtseff (Mo) number and depends on thermal conductivity, viscosity, density and specific heat capacity. The relative Mouromtseff number is defined as FOM2 and represents the ratio between the Mo number for the nanofluid and base fluid. In laminar flow, FOM2 reduces to the thermal conductivity enhancement ratio. More specifically,

(7) $$FOM2=\frac{{Mo}_{nf}}{{Mo}_{bf}}=\frac{k_{nf}}{k_{bf}}$$

where: k is the thermal conductivity, and nf and bf refer to the nanofluid and base fluid, respectively.

Another FOM is the overall heat transfer gain calculated in terms of the Nusselt number as:

(8) $$FOM3=\frac{{Nu}_{nf}}{{Nu}_{bf}}$$

In relation to overall performance, a performance evaluation criterion (PEC) can be employed as follows:

(9) $$PEC=\;\left(\frac{{Nu}_{nf}}{{Nu}_{bf}}\right)/{\left(\frac{f_{nf}}{f_{bf}}\right)}^{1/3}$$

**Figure 10:**
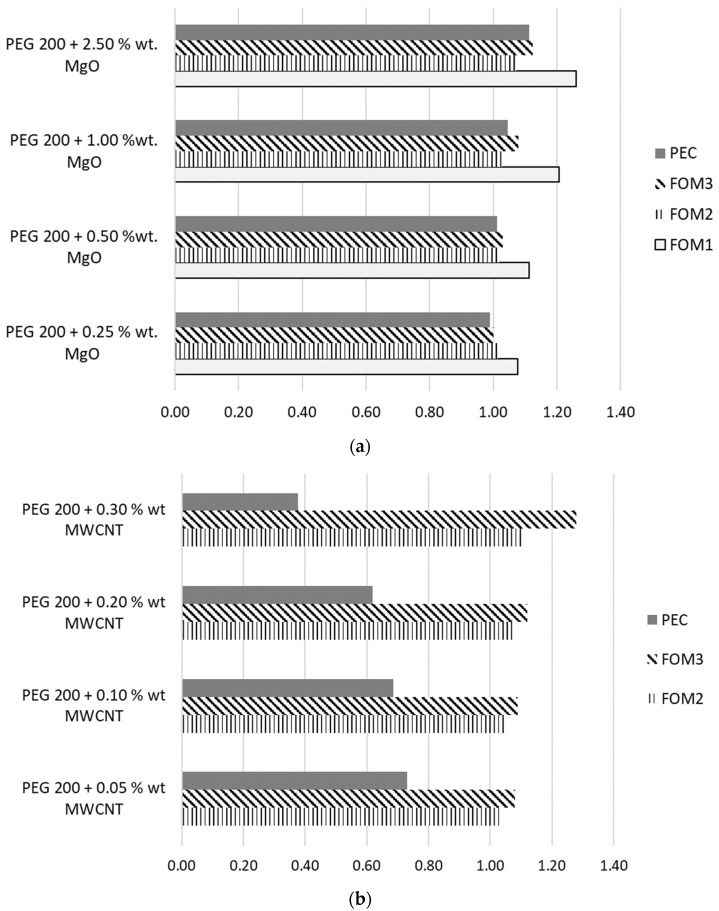
FOMs and PEC for investigated samples. (**a**) Samples with MgO. (**b**) Samples with MWCNTs.

The results for the samples investigated in the simulated flow conditions are provided in Figure 10 for one of the flow regime cases investigated here, Re = 500.

Data from Figure 10a reveals that the addition of MgO can be beneficial for heat transfer, as FOM2, FO3 and PEC are larger than one. In terms of heat transfer benefits, the addition of MgO nanoparticles results in an increase of up to 13%. On the other hand, the pumping power increases as a result of the viscosity increase, resulting in an upsurge of up to 9.5% in the skin friction coefficient. This impacts the FOM1, and this is a normal situation for all nanoparticle-enhanced fluids. On the other hand, as can be seen in Figure 10b, MWCNTs’ influence is extremely beneficial for heat transfer (i.e., very low percentages—0.3 %wt. gets a major increase in FOM3—28%); however, the PEC decreases drastically with the increase in MWCNT mass concentration. This phenomenon is brought about by the massive increase in viscosity, as was explained earlier.

## 5. Conclusions

This numerical investigation evaluated the laminar forced convection of PEG 200-based nanocolloids (Re = 500–2000) using experimentally validated properties. The study’s primary findings are as follows: Nanocolloids containing metal oxides and MWCNTs demonstrated significantly improved thermal performance compared to the base fluid, driven by superior thermal conductivity and favorable fluid–particle interactions. Common theoretical models, particularly for viscosity, tend to underestimate experimental values. Utilizing these models can lead to misleading conclusions regarding system efficiency. Assuming constant thermophysical properties during heating introduces simulation errors exceeding 10%. Incorporating temperature-dependent data is essential for high-fidelity predictive modeling. The interaction between PEG 200 and nanoparticles—especially MWCNTs—promotes complex heat transport via Brownian motion and thermophoresis. These effects are highly dependent on nanoparticle type and concentration.

In conclusion, reliable nanocolloid design requires a nuanced approach that prioritizes experimental validation and accounts for the specific chemical and rheological compatibility between the nanoparticle and the base fluid.

## Figures and Tables

**Table 1:** Chemicals properties, as per manufacturer [20].

**PEG 200 properties**	
Average Molecular Weight	190–210 g/mol
CAS Number	25322-68-3
Physical State (at 293.15 K)	Clear, colorless, viscous liquid
Melting/Freezing Point	Below 236.15 K
Density (at 293.15 K)	~1.12 g/cm^3^
Kinematic Viscosity (at 298.15 K)	3.9–4.8 mm^2^/s (cSt)
**MgO properties**	
Chemical Formula	MgO
CAS Number	1309-48-4
Crystal Structure	Cubic (Rock-salt)
Density	~3.58 g/cm^3^ (for bulk material)
Particle Size	<100 nm
Specific Surface Area	>60 m^2^/g
Melting Point	3125.15 K
**MWCNT properties**	
CAS Number	308068–56-6
Particle Size	50–90 nm diameter, 5–20 µm length, 88–100 walls
Density	2.100 g/cm^3^

**Table 2:** Regression for numerical data.

Suspension Type	Re Number	Regression	F-Statistics	*p*-Value (Approximative)	R-Squared Value
PEG 200 + MWCNT	Re = 500	h = 134.18x + 111.05	28.5	0.006	R^2^ = 0.95
	Re = 1000	h = 117.51x + 125.21	28.5	0.006	R^2^ = 0.95
	Re = 1500	h = 105.51x + 132.8	36	0.004–0.005	R^2^ = 0.96
	Re = 2000	h = 96.989x + 137.64	36	0.004–0.005	R^2^ = 0.96
PEG 200 + MgO	Re = 500	h = 9.0941x + 109.74	73.5	0.001–0.002	R^2^ = 0.98
	Re = 1000	h = 8.0573x + 122.52	148.5	<0.001	R^2^ = 0.99
	Re = 1500	h = 7.674x + 129.93	148.5	<0.001	R^2^ = 0.99
	Re = 2000	h = 7.2607x + 134.68	148.5	<0.001	R^2^ = 0.99

**Table 3:** Main characteristics of PEG 200 and PEG 400.

Base Fluid	Thermal Conductivity, W/mK	Dynamic Viscosity, Pa s	Density, kg/m^3^	Specific Heat, J/kg·K
PEG 200	0.208	0.065	1122.300	1954.000
PEG 400	0.190	0.124	1125.000	2324.775

## Data Availability

The original contributions presented in this study are included in the article. Further inquiries can be directed to the corresponding authors.

## Nomenclature

c_p_	isobaric heat capacity, J Kg^−1^ K^−1^
D	hydraulic diameter, m
h	heat transfer coefficient, W m^−2^ K^−1^
k	thermal conductivity, W m^−1^ K^−1^
L	channel length, m
P	dimensionless pressure, -
p	pressure, Pa
Pr	Prandtl number, -
r	radius, m
R	non-dimensional tube radius, -
Re	Reynolds number, -
T	temperature, K
q	wall heat flux, W m^−2^
u, v, w	velocity components, m s^−1^
u_∞_	average velocity for inlet flow, m s^−1^
U	dimensionless tangential velocity, -
V	dimensionless radial velocity, -
W	dimensionless axial velocity, -
x, y, z	cartesian coordinates, m
X, Y, Z	non-dimensional coordinates, -
Greek symbols	
θ	cylindrical coordinate, °
φ	volume concentration of nanoparticles, %
γ	shear rate, s^−1^
ρ	density, Kg m^−3^
μ	fluid dynamic viscosity, Kg m^−1^ s^−1^
Subscripts	
b	bulk
bf	base fluid
eff	effective
nf	nanocolloid
p	particle
r	“nanocolloid/base fluid” ratio
w	wall

## Abbreviations

CFD	computational fluid dynamics
CNTs	carbon nanotubes
NPs	nanoparticles
PEG	polyethylene glycol
